# Pulmonary functions of narghile smokers compared to cigarette smokers: a case–control study

**DOI:** 10.3402/ljm.v8i0.22650

**Published:** 2013-12-30

**Authors:** Helmi Ben Saad, Mehdi Khemiss, Saida Nhari, Mejda Ben Essghaier, Sonia Rouatbi

**Affiliations:** 1 Department of Physiology and Functional Explorations, Farhat Hached Hospital, Sousse, Tunisia; 2 Laboratory of Physiology, Faculty of Medicine, University of Sousse, Sousse, Tunisia; 3 Research Unit: Secondary Prevention After Myocardial Infarction, N: 04/UR/08-18, Faculty of Medicine of Sousse, Sousse, Tunisia

**Keywords:** plethysmography, tobacco, narghile, tabamel, ageing

## Abstract

**Background:**

Studies of the lung function profiles of exclusive narghile smokers (ENS) are few, have some methodological limits, and present contradictory conclusions. The present study aimed to compare the plethysmographic profiles of ENS with age- and height-matched exclusive cigarette smokers (ECS).

**Methods:**

Males aged 35–60 living in Sousse, Tunisia, who have been smoking narghile exclusively for more than 10 narghile-years (*n=*36) or cigarettes exclusively for more than 10 pack-years (*n=*106) were recruited to participate in this case–control study. The anthropometric and plethysmographic data were measured according to international recommendations using a body plethysmograph (ZAN 500 Body II, Meβgreräte GmbH, Germany). Large-airway-obstructive-ventilatory-defect (LAOVD) was defined as: first second forced expiratory volume/forced vital capacity (FEV_1_/FVC) below the lower-limit-of-normal (LLN). Restrictive-ventilatory-defect (RVD) was defined as total lung capacity < LLN. Lung hyperinflation was defined as residual volume > upper-limit-of-normal. Student *t*-test and χ^2^ test were used to compare plethysmographic data and profiles of the two groups.

**Results:**

The subjects in the ENS and ECS groups are well matched in age (45±7 *vs*. 47±5 years) and height (1.73±0.06 vs. 1.72±0.06 m) and used similar quantities of tobacco (36±22 narghile-years vs. 35±19 pack-years). Compared to the ENS group, the ECS group had significantly lower FEV_1_ (84±12 vs. 60±21%), FVC (90±12 vs. 76±18%), and FEV_1_/FVC (99±7 vs. 83±17%). The two groups had similar percentages of RVD (31 vs. 36%), while the ECS group had a significantly higher percentage of LAOVD (8 vs. 58%) and lung hyperinflation (36 vs.57%).

**Conclusion:**

Chronic exclusive narghile smoking has less adverse effects on pulmonary function tests than chronic exclusive cigarette smoking.

The past decade has seen a global increase in narghile tobacco consumption at a remarkable pace (1–4). The World Health Organization has stressed that ‘the narghile is not only a health risk, but is also a gateway to smoking for a number of young people’ (5) and the American Lung Association has described it as an ‘emerging deadly trend’ (6).

The damaging effects of narghile smoking are not well investigated (2, 7–9). To the best of our knowledge, studies of the lung function of exclusive narghile smokers (ENS) are few (10–19). These studies evaluating the chronic effects of narghile use on lung function present contradictory conclusions: lack of ventilatory defects (13, 14, 17), minimal (11) to significant (15, 16, 18) small-airways-obstructive-ventilatory-defect (SAOVD), large-airways-obstructive-ventilatory-defect (LAOVD) (10, 12, 15, 16, 18), restrictive-ventilatory-defect (RVD) (18), high frequency of lung hyperinflation (18), and acceleration of lung ageing with a significantly higher estimated-lung-age (ELA) when compared to chronological-lung-age (CLA) (18).

The majority of these studies (10–18) used limited methodology such as low sample sizes (12, 14, 17) or inclusion of mixed cigarette and narghile smokers (10) or inclusion of elderly subjects aged more than 60, who probably suffer from co-morbidities that could influence lung function (11, 15). In addition, some studies measured only expiratory flows (10–17) and not lung volumes. Furthermore, there is a lack of comparison with exclusive cigarette smokers (ECS) or healthy non-smokers (18).

A recent meta-analysis (7), which included only six studies (10–16), showed no differences in first second forced expiratory volume (FEV_1_) and in FEV_1_/forced vital capacity (FVC) between narghile and cigarette smokers. The present study aimed to compare the plethysmographic parameters, measured according to recent lung function testing guidelines (20, 21), of ENS with age- and height-matched ECS. The null hypothesis is that there is no difference between mean values of their plethysmographic data.

## Population and methods

### Type of study

This was a case–control study spread over 1 year (from February 2010 to January 2011). It was conducted in the Department of Physiology and Functional Explorations at the Farhat Hached Hospital in Sousse, Tunisia. The city lies on the Gulf of Hammamet on the Mediterranean Sea and has 173,047 inhabitants (year 2010). In unpublished data, the adjusted prevalence rates of smoking among males aged 20–93 living in Sousse were 40.1% for cigarette and 12.7% for narghile.

The study was conducted in accordance with the Declaration of Helsinki. Participants provided written consent and the study protocol was approved by the ethics committee of the hospital (approval number 2211/2009).

### Sample size

The null hypothesis (22) was *H*_0_ *: m*_1_ *=m*_2_ and the alternative hypothesis was *H*_*a*_ *: m*_1_ *=m*_2_ *+d*, where *d* is the difference between two means and *n*_1_ and *n*_2_ are the sample sizes for the ENS and ECS groups, such *N = n*_1_ *+n*_2_. The total sample size was estimated using the formula (22) *N= [(r+*1*)(Z*_*α/*2_+*Z*_1*-β*_ *)* ^2^ *σ* ^*2*^ *]/r d* ^2^. *Z*_*α*_ is the normal deviate at a level of significance = 1.96 (5% level of significance), *Z*_1*-β*_ is the normal deviate at 1-β% power with β% of type II error (0.84 at 80% statistical power); ‘*r*’ equal to *n*_1_/*n*_2_ is the ratio of sample size required for two groups (*r=*0.33 gives the sample size distribution as 1:3 for two groups). ‘*r*’ was considered because of the unequal sample sizes due to various reasons, such as higher prevalence of cigarette smokers compared to narghile smokers (of 13,776 smokers consulting a non-governmental organization (23), 34 and 11% were ECS and ENS, respectively). *σ* and *d* are the pooled standard deviation (SD) and difference of FEV_1_ means of two groups. These two values were obtained from a previous study based on a similar hypothesis (14) in which the researchers found that the mean FEV_1_ (L) in two groups was 3.2 and 3.6 and common SD was 0.85. The total sample size for the study was 142 subjects (36 ENS and 106 ECS).

### Population

Subjects were selected by convenience sampling from the staff of the local Faculty of Medicine and/or hospital, as well as from acquaintances of people involved in the study. Volunteers were not controlled for socioeconomic differences or ethnicity.

Only males aged 35–60 without a history of asthma, allergies, pulmonary tuberculosis, or recent respiratory tract infection were included. Other exclusion criteria included co-morbidities other than those related to tobacco use (e.g. cardiovascular diseases, diabetes, and neoplasia) and imperfect performance of the respiratory maneuvers. Only smokers of more than 10 narghile-years (NY, e.g. one NY for one narghile a day for 1 year) and more than 10 pack-years (PY, e.g. one PY for one pack a day for 1 year) were included in ENS and ECS groups, respectively. The ECS and ENS must have stopped smoking at least for 2 h (17, 24) and 1 day (12, 13), respectively. The type of used narghile tobacco was tabamel.

### Collected data, tobacco use evaluation, and physical examination

The following data were collected: narghile use, cigarette consumption, anthropometry [CLA, weight, height, body mass index (BMI)] and plethysmographic data [FVC, FEV_1_, FEV_1_/FVC, thoracic gas volume (TGV), residual volume (RV), total lung capacity (TLC)], and ELA.

Data were collected using a questionnaire recommended for epidemiological studies (25). Narghile use and cigarette consumption were self-reported. Height (±0.01 m) was measured with a height gauge (standing stadiometer type DETECTO^®^) with shoes removed, heels joined, and back straight. Weight (±1 kg) was measured with a digital scale (OHAUS, Florhman Park, NJ, USA) and BMI (weight/height^2^) was calculated. Depending on BMI, the participants were classified as follows (26): underweight (BMI < 18.5), normal weight (18.5 ≤ BMI <25), overweight (25 ≤ BMI <30), and obese (BMI ≥ 30).

### Plethysmographic measurements

Plethysmographic measurements were performed by MK, SN, and MBE following the American Thoracic Society and the European Respiratory Society (ATS/ERS) recommendations (20, 21). They were performed with a body plethysmograph (daily calibration) (ZAN 500 Body II, Meβgreräte GmbH, Germany).

The plethysmography procedure was explained to the participants and performed without potential harm. The plethysmograph door was closed and time was allotted for thermal transients to stabilize and for patients to relax. Subjects were then instructed to attach the mouthpiece and breathe quietly until they achieved stable end-expiration. When subjects were at or near TGV, the shutter was closed at end-expiration for 2–3 sec, and they were instructed to perform a series of gentle pants (±1 kPa) at a frequency of 0.5–1.0 Hz. A series of 3–5 technically satisfactory panting maneuvers was recorded, the shutter was opened and subjects performed an expiratory reserve volume maneuver followed by a slow inspiratory vital capacity maneuver. At least three plethysmographic TGV values that agreed within 5% were obtained and the mean value was recorded.

The FVC maneuver had three distinct phases (20): maximal inspiration, a blast of exhalation and continued complete exhalation to the end of testing. Subjects were verbally encouraged to continue exhaling air at the end of the maneuver to obtain optimal effort. The criterion for end of testing was a volume–time curve showing no change in volume (0.025 L) for 1 sec, despite the patient's effort to exhale for at least 6 sec. Repeatability was acceptable when the difference between the largest and the next largest FVC was ≤ 0.150 L and 3%, and the difference between the largest and next largest FEV_1_ was ≤ 0.150 L and 3%. Plethysmographic data were expressed in absolute values and as a percent of reference values derived from local spirometric norms (27).

Spirometric definitions were based on the recent international recommendations (20) and therefore the application of lower-limit-of-normal (LLN) and upper-limit-of-normal (ULN) (20). LAOVD was defined as a ‘FEV_1_/FVC <LLN’, RVD as ‘TLC <LLN’, mixed-ventilatory-defect (MVD) as the association of a ‘TLC <LLN’ and a ‘FEV_1_/FVC <LLN’, lung hyperinflation as ‘RV >ULN’ (28) and non-specific-ventilatory-defect (NSVD) as a decrease (<LLN) in FVC and/or FEV_1_ with normal FEV_1_/FVC and TLC (20). ELA was calculated (29).

### Statistical analysis

Variables distributions were normal and results were expressed as mean±SD.

A student's *t*-test was used to compare means of anthropometric and plethysmographic values.

A non-parametric test (Wilcoxon matched pairs test) was used to compare ELA with CLA of each group.

χ^2^ test was used to compare the profiles of the two groups.

All mathematical computations and statistical procedures were performed using Statistica software (Statistica Kernel version 6; Stat Software. France). Significance was set at the 0.05 level.

## Results

### Anthropometric data, obesity status, and tobacco use

**Fig. 1:**
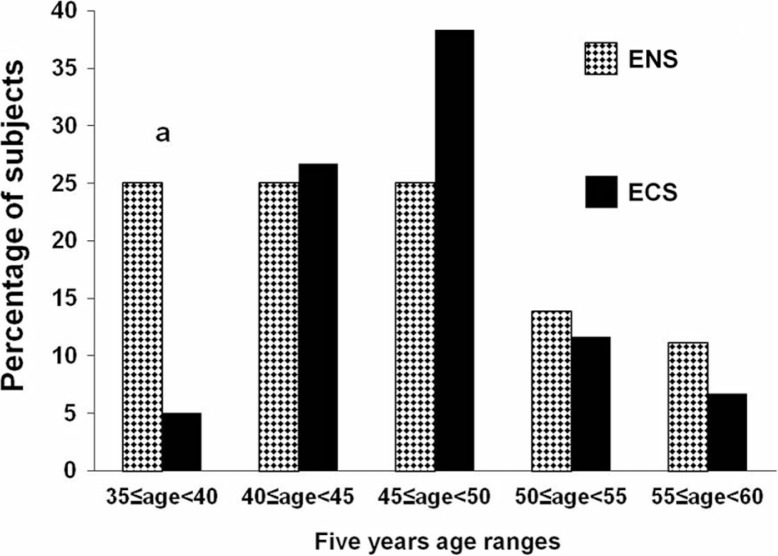
Age distribution of participants. ENS, exclusive narghile smokers; ECS, exclusive cigarette smokers. ^*a*^ *p<*0.05 (χ^2^ test): ENS vs. ECS.

Compared to ENS group, the ECS one includes low percentage of subjects aged 35–45 (25.0 vs. 5.0%, respectively) (Fig. 1).

There were no statistically significant differences between the two groups’ ages and heights (Table 1). In addition, the means of the amounts of tobacco used were similar for the two groups (36±22 NY vs. 35±19 PY, *p*=0.88). The ENS group had a significantly higher weight and BMI than the ECS group (Table 1). This reflects a tendency toward obesity as evidenced by a significantly higher percentage of obesity status in the ENS group compared to the ECS one (44.4 vs. 20.7%, respectively).

**Table 1:** Subjects’ characteristics: exclusive narghile smokers (ENS, *n*=36) and exclusive cigarette smokers (ECS, *n*=106)

		ENS	ECS	% difference
Anthropometric and spirometric data (data are mean±SD)				
Chronological-lung-age (CLA)	(yr)	45±7	47±5	−4.44
Estimated-lung-age (ELA)	(yr)	63±23#	89±34#	−41.27*
Height	(m)	1.73±0.06	1.72±0.06	0.58
Weight	(kg)	88±13	74±17	15.91*
Body mass index	(kg/m^2^)	29±4	25±5	13.79*
FEV_1_	(L)	3.39±0.58	2.41±0.88	28.91*
FEV_1_	(% pred)	84±12	60±21	28.57*
FVC	(L)	4.24±0.69	3.54±0.94	16.51*
FVC	(% pred)	90±12	76±18	15.56*
FEV_1_/FVC	(absolute value)	0.80±0.06	0.67±0.14	16.25*
FEV_1_/FVC	(% pred)	99±7	83±17	16.16*
TGV	(L)	3.93±2.16	4.35±1.53	−10.69
TGV	(% pred)	110±60	120±42	−9.09
RV	(L)	2.89±1.97	3.27±1.68	−13.15
RV	(% pred)	136±97	159±82	−16.91
TLC	(L)	7.17±2.21	7.00±1.93	2.37
TLC	(% pred)	98±30	97±26	1.02
Obesity status (data are number; percentage)				
Underweight		0 (0.00%)	7 (6.60%)	
Normal weight		3 (8.33%)	51 (48.11%)†	
Overweight		17 (47.22%)	26 (24.53%)^†^	
Obesity		16 (44.44%)	22 (20.75%)^†^	

FEV_1_, first second forced expiratory volume; FVC, forced vital capacity; RV, residual volume; TGV, thoracic gas volume; TLC, total lung capacity; Pred, predicted.

% difference=(ENS – ECS)/ENS.

* *p<*0.05 (*t*-test): ENS vs. ECS.

# *p<*0.05 (Wilcoxon test): ‘CLA’ vs. ‘ELA’ for the same group.

† *p<*0.05 (χ^2^ test): ENS vs. ESC.

### Plethysmographic parameters

The ECS group had a more pronounced tendency to an obstructive defect, with a significantly lower FEV_1_, FVC, and FEV_1_/FVC (Table 1). However, no statistically significant difference in lung volume was found between the two groups.

Narghile and cigarette smoking accelerate the aging of lung function, but this was significantly greater in the ECS group (‘ELA minus CLA’ was 42±32 vs. 18±23 years; *p*=0.001).

### Plethysmographic profiles

The percentages of subjects with plethysmographic readings outside the normal range or having ventilatory defects are shown in Table 2. The ENS group had significantly lower percentages of subjects with reduced FEV_1_ or FVC, with LAOVD, or lung hyperinflation. Percentages of subjects with RVD, MVD, or NSVD were similar in the two groups.

**Table 2:** Comparison of the plethysmographic profiles of exclusive narghile smokers (ENS, *n*=36) and exclusive cigarette smokers (ECS, *n*=106)

	ENS	ECS
Plethysmographic values lower than the lower-limit-of-normal		
Forced vital capacity (FVC)	10 (28)	55 (52)*
First second forced expiratory volume (FEV_1_)	17 (47)	93 (88)*
FEV_1_/FVC	3 (8)	61 (58)*
Total lung capacity	13 (36)	63 (31)
Plethysmographic values higher than the upper-lower-of-normal		
Thoracic gas volume	13 (36)	42 (40)
Residual volume	13 (36)	60 (57)*
Total lung capacity	13 (19)	11 (10)
Other ventilatory defects		
Mixed-ventilatory-defect	1 (3)	10 (9)
Non-specific-ventilatory-defect	7 (19)	14 (13)

Data are numbers (percentage).

* *p*<0.05 (χ^2^ test): ENS vs. ESC.

## Discussion

We compared two age- and height-matched groups: 36 ENS of more than 10 NY and 106 ECS of more than 10 PY. The ECS group had significantly lower FEV_1_, FVC, and FEV_1_/FVC, and significantly higher percentages of subjects with LAOVD or with lung hyperinflation. The two groups had similar percentages of subjects with RVD, MVD, or NSVD. Therefore, the null hypothesis, that there is no difference between the plethysmographic data of the two groups, is rejected. Both narghile and cigarette smoking accelerate the aging of lung function, but our study shows that cigarettes have a more detrimental effect.

The harmful effects of narghile use on lung function highlighted in the present study are part of a more general phenomenon (2). Studies analyzing ENS lung function are rare, have several methodological limits, and have yielded conflicting results (1, 2, 7, 18) (Supplementary file). A systematic review and meta-analysis of the chronic effects of narghile use on lung function (7) found no studies on the association of narghile smoking with airways diseases in general, and with chronic obstructive pulmonary disease (COPD) in particular.

### Comparison of plethysmographic data

Cigarette smoking could be more harmful to airways than narghile smoking (11). We found that FEV_1_, FVC and FEV_1_/FVC were significantly lower among ECS than ENS (Table 1). Our results are in agreement with other studies (FEV_1_ (10, 11), FVC (10), FEV_1_/FVC (10, 11)) but at odds with others reporting no significant differences (FEV_1_ (13), FVC (11, 13), FEV_1_/FVC (13)). Noteworthy, gender could influence comparisons (10). Al-Fayez et al. (10) reported that among females, FEV_1_ and FVC were not significantly lower among ECS than ENS. In their systematic review and meta-analysis, Raad et al. (7) concluded that the overall quality of evidence for FEV_1_ was moderate for ENS and low for ECS.

Only one study measured ENS lung volume (18). It found significant increases, in comparison with local norms, of the RV and functional residual capacity (FRC), and in the RV/TLC and FRC/TLC ratios (18). We confirm the previous data, and the increase in lung volume indicates a trend toward lung hyperinflation (Table 1). To the best of our knowledge, the present study is the first to compare ENS lung volumes with those of an ECS.

### Plethysmographic profiles

Eight percent of ENS had LAOVD and thus probably presented COPD. This percentage, close to that found by some authors (4% (11), 6% (18)), supports the idea that COPD post-narghile is more rare than post-cigarettes (11, 15, 18). This finding is contrary to other findings (30) suggesting a possible role of narghile smoking in the development of COPD (7). Indeed, we found that 58% of ECS of more than 10 PA had LAOVD. The high frequency of LAOVD among our ECS is similar to that found in a previous study on 121 ECS, of which 56% had LAOVD.

We identified RVD in 36 and 31% of ENS and ECS, respectively. A previous study found RVD in 14% of ENS of more than one NY (18). The restrictive pattern cannot be explained by the inclusion in the present study of obese subjects. Indeed, impaired respiratory function only appears in cases of severe obesity without any proven pulmonary disease (31). Moreover, in the ENS group, only three subjects presented severe obesity with an average BMI of 37±2 kg/m^2^. Thus, RVD could be explained by the particular composition of narghile smoke, which is rich in heavy metals such as lead, arsenic, and nickel (32), known as risk factors for developing fibrosis and/or pneumoconiosis (33).

We observed lung hyperinflation in 36% of ENS, which was significantly lower than observed in ECS (57%) but resembles that previously found in ENS of more than one NY (36%) (18). To the best of our knowledge, no other author has shown that narghile use is responsible for these types of defects. However, radiological studies are desirable.

#### Estimated lung-age

One of the major results of this study was that narghile use accelerates lung ageing. It was previously found that the ELA of ENS was significantly higher than CLA (47±18 vs.34±10 years) (18). However, contrary to a previous report (10) describing ENS as being at greater risk than ECS for decreased pulmonary function, we show that aging is significantly greater among ECS. Our results can be used to encourage smoking cessation (34).

### How can the harmful effects of narghile use on lung function be explained?

This question was previously asked and several hypotheses were advanced (2, 18). As in ECS (35), the answer to this question requires the study of bronchial biopsies, induced sputum samples and bronchoalveolar-lavage fluid. These explorations are rarely performed on ENS.

Recently, Nemmar et al. (36) assessed the chronic respiratory effects in mice of nose-only exposure (session of 30 min/day, 5 days/week for 1 month) to mainstream narghile smoke generated by commercially available tabamel tobacco. An increase in neutrophil and lymphocyte numbers was observed, and airway resistance was significantly increased in response to narghile smoke. Moreover, tumor necrosis factor α and interleukin 6 concentrations were significantly increased in bronchoalveolar-lavage fluid, lipid peroxidation in lung tissue significantly increased, and the level and activity of antioxidants significantly decreased. This indicates the occurrence of oxidative stress. Moreover, carboxyhemoglobin levels were significantly increased. The authors concluded that 1-month nose-only exposure to narghile smoke significantly increased airway resistance, inflammation and oxidative stress. This study (36) provides a mechanistic explanation for the detrimental chronic respiratory effects of narghile smoking. Other hypothetical explanations (Supplementary file) can be advanced (2).

#### Why narghile smoking does not affect pulmonary functions as seriously as cigarette smoking?

There are some hypotheses, advanced by Kiter et al. (11), on this issue that still need further investigation. Due to the relatively long periods of time between narghile smoking sessions in comparison to cigarette smoking, narghile use allows healing of small airways inflammation. Due to the rapid and short smoking pattern, narghile smoke does not reach the peripheral airways, and the filtration of smoke in the water may play the most important role in decreasing the harmful effects (11). Carbon monoxide (CO), nicotine, and tar, the most harmful contents of tobacco smoke, are filterable. However, recent evidence has shown that water does not significantly filter out the nicotinic products in narghile smoke (37).

Highlights of studies on the chronic effects of narghile smoking on lung function are presented in the Supplementary file. Only some studies (13, 15, 16, 18) used standardized medical questionnaires. There is an urgent need to standardize items in epidemiological questionnaires used in studies on narghile use (38). The present study applied non-inclusion criteria, discussed in the Supplementary file, are in line with these reported for such studies (10–19). The present study high percentage of obese ENS (two times more than ECS) is similar to that reported by Shafique et al. (39) (i.e. compared to healthy non-smokers, ENS were significantly more likely to have obesity; odds ratio = 1.93). In addition, the deterioration in lung function associated with obesity appears only in the case of severe obesity without any proven pulmonary disease (31). In the present study, only 6% of the ENS presented severe obesity; they were not excluded. Other factors not assessed in our study, such as occupational exposure, environmental pollutants, socioeconomic factors, or diet, might have influenced the results. However, when answering the medical questionnaire, no ENS reported being followed by an occupational physician. The calculated sample size (*n*=142) is satisfactory (22). It is larger than those used in some studies (12, 14, 17) but smaller than in others (10, 11, 13, 15, 16, 18). Unlike most other studies, we measured static lung volumes, which are essential to diagnosis RVD (20) and lung hyperinflation (40). Contrary to most published studies, which used old spirometric recommendations and definitions, we applied recent international definitions (20) based on the use of LLN and ULN notions. In addition, unlike two other studies (11, 15) that applied Caucasian norms, we used Tunisian spirometric reference values (27). The application of inappropriate norms leads to misinterpretation of spirometry data in a substantial proportion of subjects, which could result in an inaccurate diagnosis (41). It is important to note that some studies used neither spirometric guidelines (10, 12, 17) nor spirometric norms (10, 12–14, 16, 17).

### Study limitations

Convenience sampling is a statistical method of drawing representative data by selecting people because of the ease of their volunteering. Its advantages are the availability and the quickness with which data can be gathered. Its disadvantages are the risk that the sample might not represent the population as a whole, and it might be biased by volunteers.

Smoking cessation of 1 day for narghile and 2 h does not seem to affect the results. In fact, according the ERS/ATS (42), smoking within at least 1 h of testing is an activity that should preferably be avoided prior to lung function testing. In addition, the first changes associated with smoking are in the small airways (43) and so the duration since the last smoking affects only the prevalence of SAOVD.

In this study, we analyzed the chronic effects of exclusive narghile use on respiratory impairment using plethysmographic markers. However, there are other markers, such as CO in expired air or carboxyhemoglobin, the study of respiratory mucociliary clearance half-life, the percentage of retention of radioactivity at the end of the first hour, or penetration index (12, 14, 44, 45). Completion of the evaluation with a bronchial reversibility test (46) and/or measurement of diffusing capacity for CO (47) would also have been useful. Similarly, it would have been useful to perform, as part of disability and handicap evaluation, a walk test and a quality of life questionnaire (48). In this study, we focused only on the purely functional aspects of exclusive narghile use. However, ENS may complain of some clinical signs, such as dyspnea, wheezing, or respiratory illness (8, 13, 15, 16).

In conclusion, chronic exclusive narghile smoking is less detrimental to pulmonary function than chronic exclusive cigarette smoking.
